# Transient Overexpression of *VvMYBPA1* in Grape Berries Enhances Susceptibility to *Botrytis cinerea* Through ROS Homeostasis Modulation

**DOI:** 10.3390/plants14162469

**Published:** 2025-08-09

**Authors:** Lihong Hao, Yuxin Zhang, Zeying Ge, Xinru Meng, Yu Sun, Huilan Yi

**Affiliations:** School of Life Science, Shanxi University, Taiyuan 030006, China; zhangyuxin39@sxu.edu.cn (Y.Z.); gezeying@sxu.edu.cn (Z.G.); mengxinru@sxu.edu.cn (X.M.); sunyu3@sxu.edu.cn (Y.S.)

**Keywords:** *Botrytis cinerea* stress, grape, MYBPA1, ROS, proanthocyanidin

## Abstract

Gray mold disease, caused by *Botrytis cinerea*, severely impacts grape production worldwide. Although proanthocyanidins (PAs) contribute to fungal pathogen resistance, their role in grape defense against *B. cinerea* remains unclear. Here, we demonstrate that VvMYBPA1, a key transcriptional regulator of PA biosynthesis, negatively modulates *B. cinerea* resistance in grape berries. While infection suppressed endogenous VvMYBPA1, its agroinfiltration-mediated transient overexpression in berries elevated susceptibility, paralleling reduced β-1,3-glucanase (BGL) and polyphenol oxidase (PPO) activities. Additionally, VvMYBPA1 overexpression elevated *VvRBOHs’* expression and reduced peroxidase (POD) activity, resulting in excessive hydrogen peroxide (H_2_O_2_) accumulation and more cell death. Our results reveal that VvMYBPA1 negatively regulates *B. cinerea* resistance by disrupting antioxidant enzyme activity and ROS homeostasis, providing new insights into the interplay between PA biosynthesis and fungal defense mechanisms.

## 1. Introduction

Grape (*Vitis vinifera* L.) is a globally vital fruit crop with high economic impact but its production faces severe threats from multiple diseases, notably *Botrytis cinerea*-induced gray mold [1]. *B. cinerea* is a broad-host-range necrotrophic fungal pathogen, causing significant pre- and post-harvest losses in grape berries [2,3]. The grapefruit-*B. cinerea* pathosystem exhibits a dynamic molecular interplay characterized by host pattern-triggered immunity and fungal effector-mediated virulence [4]. Elucidating the molecular basis of this interaction is essential for developing grape resistance strategies and mitigating economic impacts.

*B. cinerea* triggers lipid peroxidation and ROS bursts in host tissues [5]. ROS exhibit dual functionality in plant–pathogen systems [6]. ROS exhibit a biphasic role: at low levels, they mediate defense signaling (hypersensitive response, callose deposition); at high levels, they provoke oxidative injury, aiding necrotrophy proliferation [5,7]. The homeostatic regulation between ROS generation and detoxification is tightly regulated by a network of enzymes, including NADPH oxidases (RBOHs), peroxidase (POD), superoxide dismutase (SOD), and catalase (CAT). Emerging evidence underscores the pivotal regulatory role of ROS homeostasis in plant defense against *B. cinerea*. Molecular studies have demonstrated that Sly-miR398b-mediated disruption of ROS equilibrium compromises tomato resistance to this pathogen [8]. Similarly, genetic evidence confirms SlWRKY46 as a pivotal transcriptional controller of *B. cinerea* resistance through ROS metabolism reprogramming [9]. Supporting these findings, a comparative transcriptomic study between resistant and susceptible grape cultivars uncovered that perturbations in ROS metabolism and redox regulation significantly influence host susceptibility [10]. In grape berries, the regulation of ROS homeostasis during *B. cinerea* infection remains poorly understood, particularly in the context of transcriptional regulators such as MYB transcription factors.

MYB transcription factors play critical roles in plant secondary metabolism, stress responses, and defense mechanisms [11]. Among them, VvMYBPA1 has been functionally characterized as a transcriptional regulator of proanthocyanidin (PA) biosynthesis in grapevine through direct activation of anthocyanidin reductase (ANR) and leucoanthocyanidin reductase (LAR) promoters [12]. PAs, or condensed tannins, significantly influence fruit taste and wine flavor, underscoring the economic importance of their biosynthesis regulation. Research shows PAs also play key roles in plant–pathogen interactions. For example, cucumber CsMYB60 enhances resistance to *Fusarium solani* by promoting PA synthesis [13], while poplar MYB115 directly activates PA-specific genes *ANR1* and *LAR3*, boosting resistance to *Dothiorella gregaria* [14]. In grapevines, the VvPUB26-VvWRKY24-VvDFR/VvLAR and VqWRKY56-VqbZIPC22-VvCHS3/VvLAR1/VvANR modules regulate PA biosynthesis, enhancing leaf resistance to *Erysiphe necator* [15,16]. Additionally, *V. amurensis* VvbHLH137 increases fruit resistance to *Colletotrichum gloeosporioides* by promoting PA accumulation [17]. Emerging evidence demonstrates that MYB transcription factors can influence ROS production and scavenging, but the underlying mechanisms remain elusive. For instance, besides the role in the biosynthesis of anthocyanidin, SlMYB75 improved the scavenging of excess H_2_O_2_ to resist *B. cinerea*, suggesting a potential trade-off between secondary metabolite biosynthesis and ROS-mediated defense responses [18]. Despite its known functions, VvMYBPA1’s precise role in orchestrating ROS metabolism and immune responses against *B. cinerea* infection has not been systematically elucidated.

In this study, we systematically examined VvMYBPA1’s mechanistic involvement in orchestrating ROS metabolism and defense activation during *B. cinerea* pathogenesis, using both constitutively overexpressing *Arabidopsis thaliana* and agroinfiltration-mediated transient overexpression in grape berries. Our findings not only advance the knowledge of MYB transcription factors in plant–pathogen interactions but also offer potential strategies for enhancing grape resistance to *B. cinerea* through targeted manipulation of ROS dynamics and secondary metabolism.

## 2. Results

### 2.1. Overexpression of VvMYBPA1 Enhances the Susceptibility of Arabidopsis Leaves to B. cinerea

**Figure 1:**
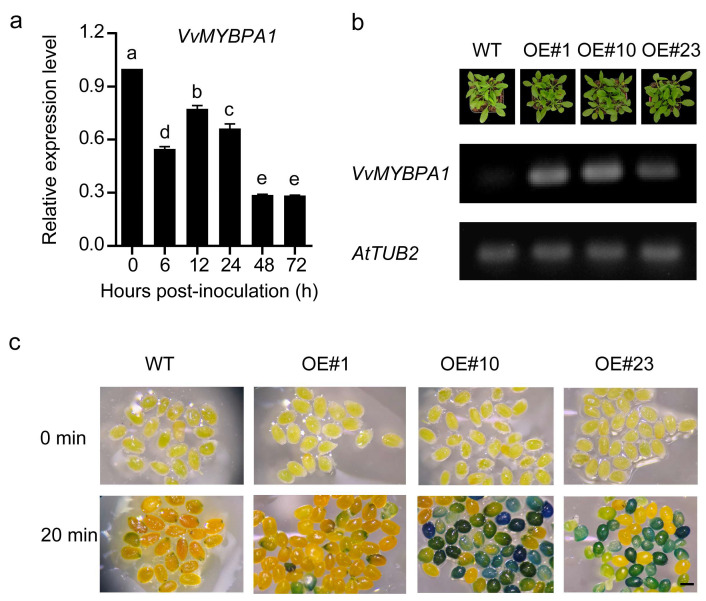
Heterologous overexpression of *VvMYBPA1* in *Arabidopsis thaliana*. (**a**) Temporal expression profile of *VvMYBPA1* in grape berry skins following *B. cinerea* infection. Different letters denote significant differences among groups (one-way ANOVA, *p* < 0.05). (**b**) Relative expression of *VvMYBPA1* in WT and three independent transgenic Arabidopsis lines (OE#1, OE#10, OE#23), normalized to *AtTUB2*. (**c**) Proanthocyanidin accumulation in seeds visualized by DMACA staining. Scale bar = 1 mm.

**Figure 2:**
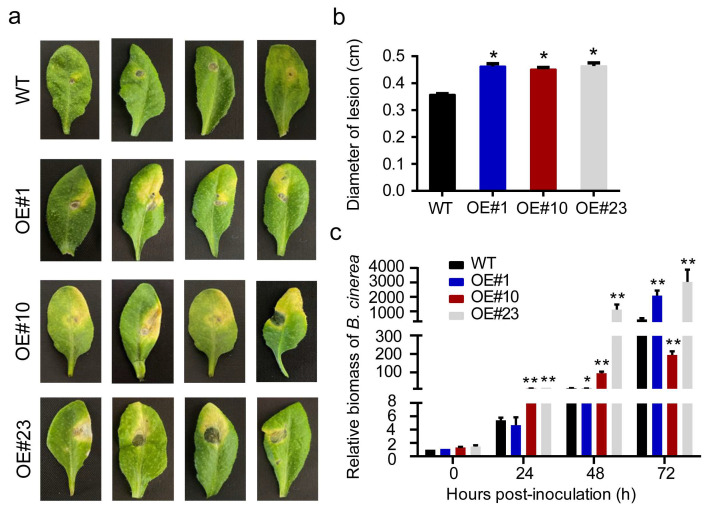
Ectopic expression of VvMYBPA1 enhances Arabidopsis sensitivity to *B. cinerea* infection. (**a**) Leaf symptoms observed in WT and three independent VvMYBPA1-overexpressing lines (#1, #10, #23) at 72 hpi. (**b**) Quantification of necrotic lesions on WT and VvMYBPA1-OE Arabidopsis leaves at 72 hpi. Data represent mean lesion diameters from three independent experiments (*n* = 33). (**c**) *B. cinerea* colonization was monitored by quantifying the ratio of *BcActin* to *AtActin* gene amplification in infected leaf samples collected at different infection stages. Error bars denote standard error of triplicate experiments. Statistical comparisons were made using two-tailed *t*-tests (* *p* < 0.05, ** *p* < 0.01).

To investigate the role of PA biosynthesis in resistance to *B. cinerea*, we first analyzed VvMYBPA1 expression in mature ‘Kyoho’ grape berries at harvesting stage (E-L 38) during infection (Figure 1a). At 6 hpi, VvMYBPA1 expression decreased by nearly half, and by 48 hpi, it dropped to less than two-thirds of the initial level. To further explore cultivar-specific responses, we analyzed VvMYBPA1 expression across different varieties using the Botrytis Stress Atlas Explorer. While ‘Pinot’, ‘Semillon’, and ‘Garganega’ (G) berries showed negligible VvMYBPA1 induction upon infection, ‘Furmint’ exhibited progressive upregulation correlating with disease severity, suggesting a negative association between VvMYBPA1 expression and resistance (Figure S1) [19,20]. To elucidate VvMYBPA1’s involvement in defense mechanisms, we established overexpression Arabidopsis lines, from which three transgenic T3 progenies (OE#1, OE#10, and OE#23) showing stable transgene expression were isolated (Figure 1b). DMACA staining of immature seeds revealed that the transgenic lines, particularly OE#10 and OE#23, exhibited intense blue staining within 20 min, indicating higher PA accumulation relative to wild-type controls (Figure 1c). Pathogenicity assays using detached leaves revealed significantly enhanced susceptibility to *B. cinerea* in transgenic lines relative to WT plants at the 4-week developmental stage, including increased leaf yellowing and larger lesion diameters at 72 hpi (Figure 2a,b). Furthermore, quantification of fungal colonization confirmed higher *B. cinerea* levels in the transgenic lines (Figure 2c). These results demonstrate that VvMYBPA1 overexpression enhances Arabidopsis susceptibility to *B. cinerea*.

### 2.2. VvMYBPA1 Stimulates ROS Production in A. thaliana Leaves upon B. cinerea Challenge

**Figure 3:**
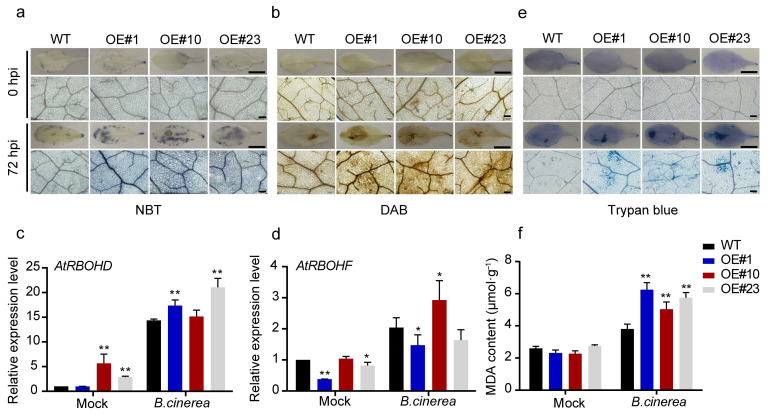
VvMYBPA1 overexpression exacerbates pathogen-triggered ROS production in Arabidopsis. (**a**) NBT staining reveals O_2_^−^ distribution. (**b**) DAB staining indicates H_2_O_2_ accumulation. Analyses performed on WT and three independent VvMYBPA1-expressing lines at 72 hpi. Scale bars: 1 cm (whole leaf), 200 µm (magnified view). (**c**,**d**) Expression profiles of *AtRBOHD* and *AtRBOHF* in WT and transgenic lines during infection (0 and 72 hpi), normalized to *AtActin2.* (**e**) Trypan blue staining reveals cell death patterns at 72 hpi. Scale bars as in A-B. (**f**) MDA content as a lipid peroxidation marker at 0 and 72 hpi. Values shown are means ± SE from three biological replicates. Statistical significance was assessed by two-tailed Student’s *t*-test (* *p* < 0.05, ** *p* < 0.01).

Given the critical role of ROS generation in plant interactions with *B. cinerea*, we assessed H_2_O_2_ and O_2_^−^ accumulation in Arabidopsis leaves 72 hpi using DAB and NBT staining. In contrast to the wild-type (WT) controls, the overexpressing lines exhibited darker staining, indicating elevated ROS levels (Figure 3a,b). In *Arabidopsis thaliana*, the NADPH oxidase family members AtRBOHD and AtRBOHF serve as primary enzymatic sources of ROS generation [21]. We analyzed their transcription levels in overexpression lines and WT plants post inoculation. At 72 hpi, *AtRBOHD* expression was significantly higher in OE#1 and OE#23 compared to WT, while *AtRBOHF* transcript levels were markedly elevated in OE#10 (Figure 3c,d). Additionally, malondialdehyde (MDA) levels, indicative of ROS-mediated lipid peroxidation [22], were substantially higher in transgenic lines than in WT after inoculation (Figure 3f). Trypan blue staining further revealed increased cell death in transgenic leaves compared to WT at 72 hpi (Figure 3e). These findings suggest that in Arabidopsis VvMYBPA1 promotes *B. cinerea* proliferation by inducing oxidative burst-mediated cell death.

### 2.3. VvMYBPA1-Overexpressing Grape Berries Exhibit Decreased Resistance to B. cinerea Infection

**Figure 4:**
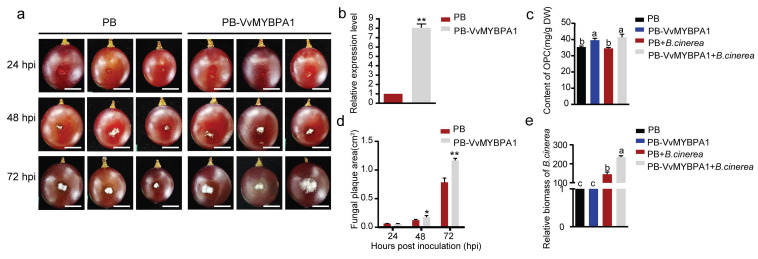
Transient overexpression of VvMYBPA1 increases susceptibility of ‘Kyoho’ grape berries to *B. cinerea* infection. (**a**) Disease symptoms on grape berries infiltrated with either empty vector (PB) or VvMYBPA1 overexpression construct (PB-VvMYBPA1) at 24, 48, and 72 h post inoculation with *B. cinerea* (spore suspension: 2 × 10^6^/mL). Representative images shown were derived from three biological replicates (*n* = 30). Bars = 1 cm. (**b**) Relative expression of VvMYBPA1 in transiently transformed berries. (**c**) Oligomeric proanthocyanidin (OPC) content in PB and PB-VvMYBPA1 berries at 0 and 72 hpi. (**d**) Lesion area quantification at 24, 48, and 72 hpi (*n* = 30 from three independent experiments). (**e**) *B. cinerea* biomass was quantified via qPCR at 0 and 72 hpi. Bars represent mean ± SE. Significant variations between treatments marked by * (*t*-test: * *p* < 0.05, ** *p* < 0.01) or letters (ANOVA, *p* < 0.05).

Assessing the defensive function of VvMYBPA1 in grape berry–pathogen interactions, we generated transient VvMYBPA1-overexpressing grape berries via Agrobacterium-mediated transformation and inoculated them with *B. cinerea* (Figure 4a). VvMYBPA1 expression in transgenic berries was 8-fold higher than in controls, accompanied by significantly elevated proanthocyanidin levels both pre- and post-inoculation (Figure 4b,c). Three days post inoculation, lesion areas in the overexpression group were significantly larger, and *B. cinerea* colonization was markedly higher compared to controls (Figure 4d,e). These results, consistent with heterologous overexpression in Arabidopsis, demonstrate that VvMYBPA1 overexpression compromises grape berry resistance against *B. cinerea*.

**Figure 5:**
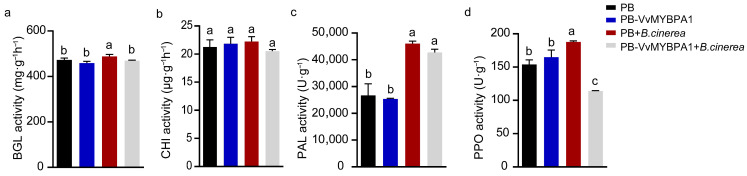
VvMYBPA1 overexpression alters defense enzyme responses to *B. cinerea* infection. (**a**–**d**) Activities of four defense-related enzymes (BGL, CHI, PAL, PPO) measured at 72 hpi. Data shown as mean ± SE from three biological replicates. Different letters denote statistical differences (ANOVA, *p* < 0.05).

We further monitored the enzymatic activity of defense-associated proteins spanning both phenylpropanoid pathway (PAL, PPO) and pathogen cell wall degradation (BGL, CHI) functions. Prior to *B. cinerea* inoculation, the enzymatic profiles of VvMYBPA1-overexpressing and control berries showed comparable activity levels (Figure 5a–d). Post inoculation, besides CHI activity, all other three enzyme activities significantly increased in the control berries (Figure 5a–d). In contrast, the VvMYBPA1-overexpression group exhibited distinct responses; while PAL and CHI activities showed comparable activities compared to the control (Figure 5b,c), BGL and PPO activities were significantly reduced (Figure 5a,d). These findings further demonstrate that VvMYBPA1 overexpression compromises grape berry resistance against *B. cinerea* infection.

### 2.4. VvMYBPA1 Promotes ROS Accumulation in Mature Grape Berries During B. cinerea Inoculation

**Figure 6:**
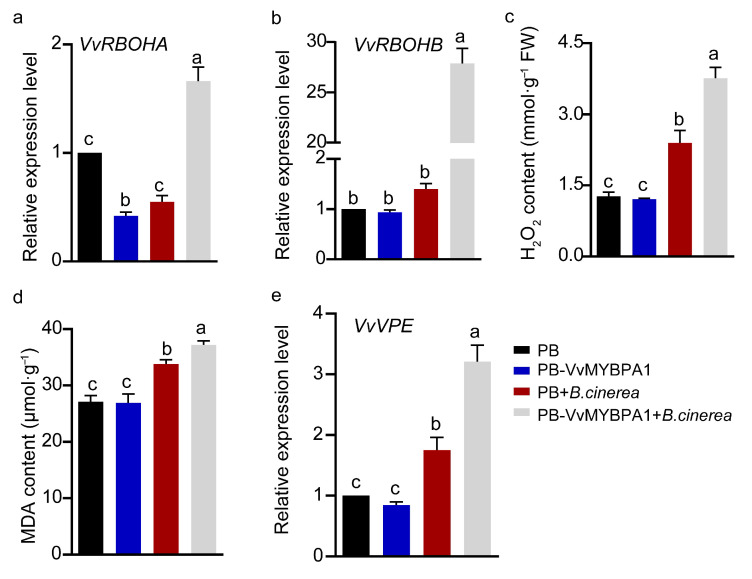
VvMYBPA1 overexpression alters *B. cinerea*-induced ROS accumulation in grape berries. (**a**,**b**) Expression profiles of NADPH oxidase genes (*VvRBOHA* and *VvRBOHB*) in control (PB) and VvMYBPA1-overexpressing (PB-VvMYBPA1) grape berries at 0 and 48 hpi. *Vvβactin* served as the internal reference gene. (**c**,**d**) H_2_O_2_ and MDA levels in PB and PB-VvMYBPA1 berries at 0 and 72 hpi. (**e**) Relative expression of *VvVPE* in PB and PB-VvMYBPA1 berries at 0 and 48 hpi, normalized to *Vvβactin*. Values are means ± SE of three biological replicates. Distinct letters indicate ANOVA-grouped differences (*p* < 0.05).

To further investigate whether VvMYBPA1 in grape berries promotes *B. cinerea* proliferation by enhancing ROS accumulation, we analyzed the expression levels of two key *VvRBOH* genes, *VvRBOHA* and *VvRBOHB*, in VvMYBPA1-overexpressing and control berries two days post inoculation. The results revealed that both genes were significantly upregulated in the overexpression group relative to the control, with *VvRBOHB* expression approximately 18-fold higher (Figure 6a,b). Additionally, hydrogen peroxide (H_2_O_2_) levels were measured three days post inoculation. While no significant difference was observed prior to inoculation, H_2_O_2_ content in the overexpression group was markedly higher (approximately 1.5-fold) than in the control group post inoculation (Figure 6c). Furthermore, MDA levels were significantly elevated in the overexpression group (Figure 6d). VvVPE, a crucial enzyme in programmed cell death that facilitates vacuole rupture and subsequent release of hydrolytic enzymes, was also examined [23]. Its expression was low and comparable between groups before inoculation but increased approximately 2-fold in the overexpression group post inoculation (Figure 6e). These findings collectively demonstrate that VvMYBPA1 overexpression enhances H_2_O_2_ accumulation, exacerbates membrane lipid peroxidation, promotes host cell death, and ultimately facilitates *B. cinerea* proliferation.

### 2.5. Overexpression of VvMYBPA1 Altered the Activities of Antioxidant Enzymes in Grape Berries

**Figure 7:**
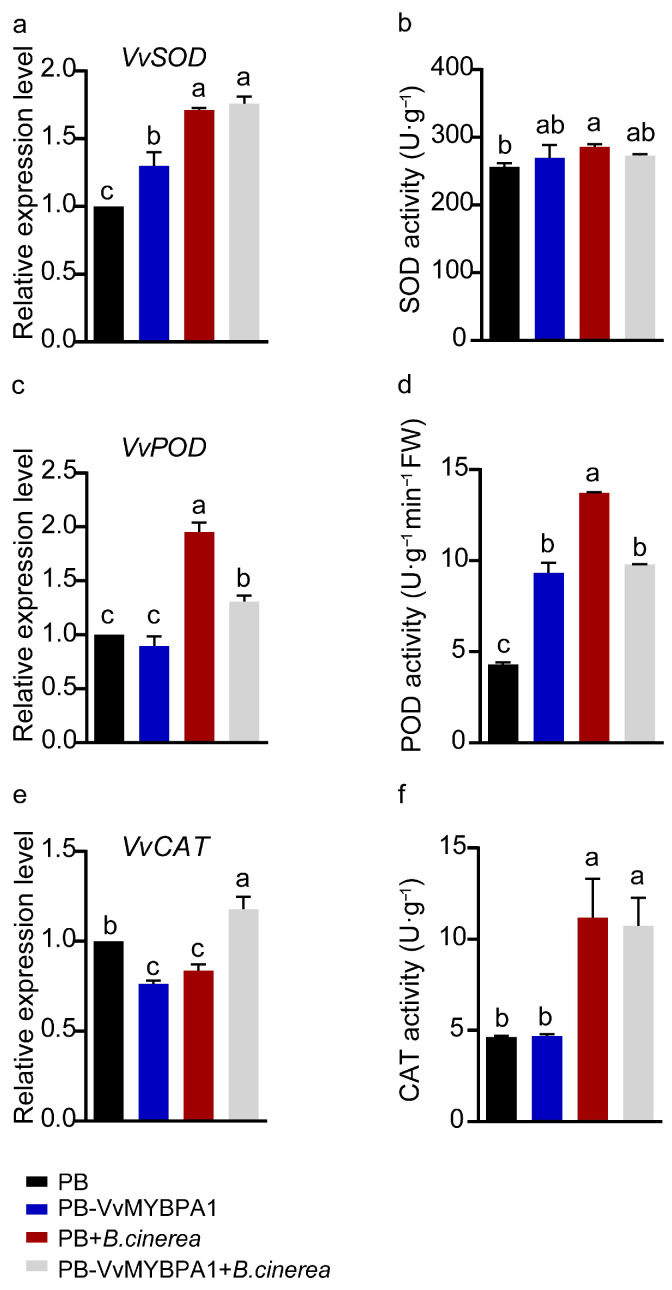
Transcriptional and enzymatic reprogramming of antioxidant systems in transgenic berries during *B. cinerea* infection. (**a**,**b**) SOD dynamics. (**c**,**d**) POD parameters. (**e**,**f**) CAT profiles. Transcriptional changes (assessed at 48 hpi) and enzymatic alterations (evaluated at 72 hpi) are shown. Bars show mean ± SE of three biological replicates. Letter-based significance groupings (ANOVA, *p* < 0.05).

To elucidate the dual role of VvMYBPA1 in ROS metabolism during grape–*B. cinerea* interaction, we measured the transcript levels of key antioxidant enzymes (SOD, POD, and CAT) at 48 hpi and their corresponding enzyme activities at 72 hpi in both VvMYBPA1-overexpressing and control berries. Transcriptional analysis revealed a pathogen-induced upregulation of SOD in both genotypes, with no significant variation between genotypes (Figure 7a). Consistently, SOD activity remained comparable between transgenic and control fruits (Figure 7b). Notably, VvMYBPA1 overexpression led to a marked suppression of both POD transcription and enzymatic activity compared to the control (Figure 7c,d). While CAT transcript accumulation was enhanced in transgenic berries, its catalytic activity showed no significant alteration (Figure 7e,f). These findings demonstrate that VvMYBPA1 orchestrates antioxidant enzyme regulation during *B. cinerea* infection, extending beyond its role in ROS biosynthesis in grape berries.

## 3. Discussion

### 3.1. VvMYBPA1 Negatively Regulates B. cinerea Resistance in Mature Grape Berries

*B. cinerea*, a highly destructive fungal pathogen, poses a significant threat to the post-harvest storage of grape berries. Understanding the defense mechanisms against gray mold is therefore crucial for improving grape quality and extending shelf life during storage. Numerous secondary metabolites in plants contribute to fungal pathogen resistance, among which PAs play a pivotal role. For instance, heterologous expression of MnANR and MnLAR from *Morus alba* in *Nicotiana tabacum* has been demonstrated to confer enhanced defense against *B. cinerea* infection [24]. Additionally, catechin-treated cucumber seedlings exhibit increased pathogen resistance [13]. Previous investigations have well established the contribution of PAs to *Vitis vinifera* defense mechanisms against *Erysiphe necator* infection. For example, VvPUB26 positively regulates resistance by ubiquitinating and degrading the PA synthesis repressor VvWRKY24 in grape leaves. Interestingly, VvPUB26-silenced plants show reduced powdery mildew resistance but elevated PA accumulation [15]. Genetic evidence from *Vitis quinquangularis* reveals that the VqWRKY56-VqbZIPC22 transcriptional module activates proanthocyanidin biosynthesis pathways, resulting in improved defense against *Erysiphe necator* infection [16].

The proanthocyanidin biosynthetic pathway requires coordinated action of multiple enzymes, including chalcone synthase catalyzing the initial condensation, chalcone isomerase mediating flavonoid ring formation, dihydroflavonol reductase reducing dihydroflavonols, and the terminal reductases LAR and ANR generating different PA subunits [24]. Several transcription factors, including VvMYBPAR, VvMYBPA1, VvMYBPA2, VvMYB5b, and VviMYB86, have been functionally identified as major determinants of PA biosynthesis in grapevines [12,25,26,27,28]. As previously reported, PA content in grape berry skins, along with the transcriptional level of *VvMYBPA1*, *VvLAR*, and *VvANR*, declines during berry ripening [12,29]. In this study, we investigated the function of VvMYBPA1 in gray mold resistance by ectopically expressing it in Arabidopsis and transiently overexpressing it in mature grape berries (Figure 2 and Figure 4). Surprisingly, VvMYBPA1 overexpression in mature berries increased PA accumulation but significantly reduced resistance to *B. cinerea*. These findings expand the knowledge of the complex relationship between PA biosynthesis and gray mold resistance.

Plants employ two key immune strategies against pathogens: pattern-triggered immunity (PTI) and effector-triggered immunity (ETI) [30]. Upon *B. cinerea* infection, pathogen-associated molecular patterns are recognized, triggering host defenses such as transcriptional reprogramming, PR protein secretion, ROS burst, and activation of secondary metabolic pathways [4,10]. Key enzymes, including BGL, CHI, PAL, and PPO, play critical roles [31,32]. BGL and CHI degrade fungal cell walls, leading to pathogen death. PAL, central to the phenylpropanoid pathway, enhances lignin and flavonoid synthesis when activated. In grapes, 15 *PAL* genes contribute to *B. cinerea* resistance during the green berry stage [33,34]. PPO promotes lignification and generates antimicrobial quinones via phenolic oxidation [35]. In our study, pre-inoculation enzyme activities showed no significant differences between VvMYBPA1-overexpressing and control fruits. Post inoculation, however, enzyme activities declined in overexpressing fruits, with significant reductions in BGL and PPO (Figure 5). This suggests VvMYBPA1 negatively regulates *B. cinerea* resistance by suppressing key defense enzyme activities in secondary metabolism.

### 3.2. ROS and PAs in Plant Defense: A Dual Role

ROS are pivotal signaling molecules that mediate plant defense mechanisms against diverse environmental challenges, especially in immune responses. Studies have demonstrated that ROS production is closely associated with both PTI and ETI [36]. As secondary messengers, ROS can induce hypersensitive responses, ultimately leading to host cell death. This mechanism effectively restricts nutrient acquisition by biotrophic pathogens, thereby inhibiting their proliferation [37,38,39]. Nevertheless, an overabundance of ROS can trigger programmed cell death in host tissues, rendering plants more vulnerable to infection by hemi-biotrophic and necrotrophic pathogens [40,41]. Research has shown that in the interaction with the hemi-biotrophic pathogen *Phytophthora infestans*, the tomato 1R-type MYB transcription factor SlKUA1 enhances *SlRBOHD* expression, increasing ROS accumulation, while simultaneously reducing POD expression by binding to the *SlPrx1* promoter, thereby impairing ROS scavenging and ultimately affecting tomato resistance to late blight [41]. In contrast, during interaction with the necrotrophic pathogen *B. cinerea*, tomato SlWRKY3 promotes ROS accumulation and negatively regulates resistance by affecting *SlCAT1* expression [40]. Consistent with these findings, our study detected elevated ROS levels in both VvMYBPA1-overexpressing Arabidopsis leaves and grape fruits following *B. cinerea* infection (Figure 3 and Figure 6), accompanied by significantly reduced POD expression and activity (Figure 7). The disruption of ROS homeostasis ultimately led to increased MDA content, elevated host cell death, and upregulation of the apoptosis-related gene *VvVPE*, which facilitated *B. cinerea* proliferation (Figure 6).

PAs, as major defensive phenolic compounds, exhibit antioxidant activity [42]. However, our study revealed that VvMYBPA1 overexpression not only increased proanthocyanidin content (Figure 1 and Figure 4) but also enhanced ROS accumulation (Figure 3 and Figure 6), affecting the expression and activity of antioxidant enzymes such as CAT, POD, and SOD (Figure 7), ultimately exacerbating membrane lipid peroxidation and host cell death (Figure 3 and Figure 6). Parallel results have been documented in previous research. The WRKY transcription factor VqWRKY56, identified in wild grape *Vitis quinquangularis*, promotes anthocyanin and proanthocyanidin accumulation in grape leaves through interaction with VqbZIP22. Following powdery mildew infection, VqWRKY56-overexpressing lines exhibited elevated ROS levels and increased leaf cell death, which significantly inhibited fungal hyphal growth and spore production [16]. These findings unveil a sophisticated interplay between proanthocyanidin accumulation and defense activation during infection, where these processes exhibit dynamic and context-dependent coordination.

**Figure 8:**
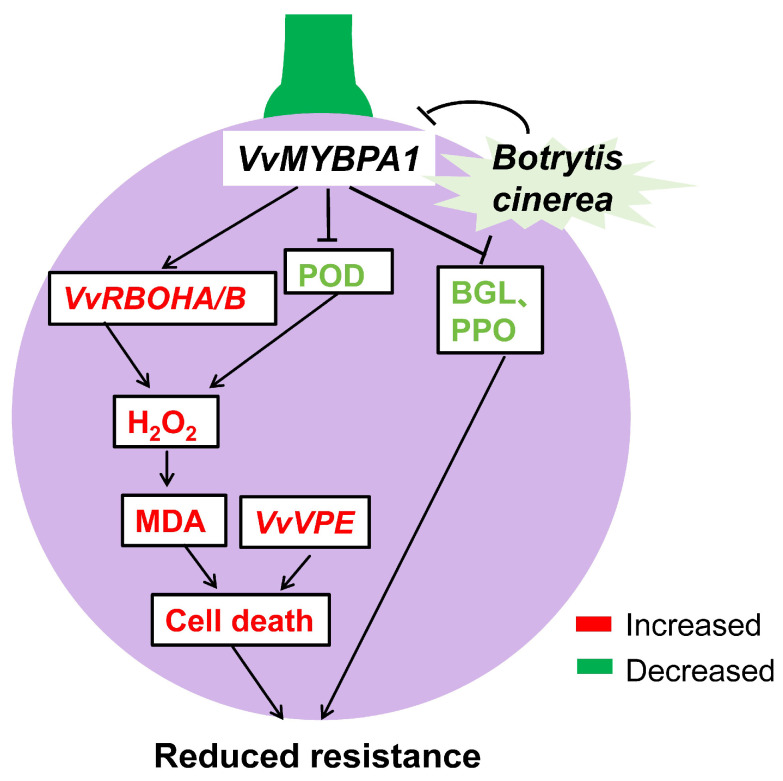
A proposed model illustrating how *VvMYBPA1* negatively regulates *Botrytis cinerea* resistance by modulating ROS homeostasis.

This work characterizes VvMYBPA1’s function in mediating the grape–*B. cinerea* interaction, with particular emphasis on its role in maintaining ROS balance (Figure 8). We demonstrated that transient overexpression of VvMYBPA1 in berries enhances ROS accumulation and lipid peroxidation, increasing susceptibility to *B. cinerea*. The transient overexpression of MYBPA1, while capable of improving the content of PAs, concomitantly attenuates the established defense mechanisms in ripening grape berries against *B. cinerea* pathogenesis. This research significantly contributes to comprehending the intricate interplay between secondary metabolism and oxidative stress in grape–pathogen interactions. Future work will employ CRISPR-Cas9 knockout or RNAi-mediated knockdown of VvMYBPA1 in stable transgenic grape calli to definitively establish its negative regulatory role in Botrytis defense responses. These genetic studies will not only elucidate the molecular mechanism of susceptibility but also provide direct targets for breeding Botrytis-resistant grape cultivars through precision genome editing of MYB transcription factors.

## 4. Materials and Methods

### 4.1. Plant Materials and Growth Conditions

*Arabidopsis thaliana* ecotype Columbia (Col-0) was maintained under laboratory conditions. Seeds were surface-sterilized in 15% (*v*/*v*) NaClO and then were treated at 4 °C for 3 days on half-strength Murashige and Skoog (MS) medium. Wild-type and transgenic seedlings were then transferred into an autoclaved soil mix at the two-leaf stage. Plants were cultivated in a controlled-environment growth chamber (22 °C, 16/8 h photoperiod, 60% RH). For this study, *Vitis vinifera* cv. ‘Kyoho’, a cultivar reported to show resistance to *B. cinerea* [43] and widely cultivated in Shanxi Province, was sampled from an orchard in Taiyuan City (112°33′ E, 37°51′ N). Post harvest, berries at E-L 38 stage were promptly transported to the laboratory for transient transformation and *B. cinerea* pathogenicity assays.

### 4.2. Vector Construction

To clone the *VvMYBPA1* gene into an overexpression vector, the VvMYBPA1 open reading frame (ORF) was PCR-amplified from cDNA derived from grape skins and VvMYBPA1 CDS F/VvMYBPA1 CDS R as primers and then cloned into pDONR221 (Invitrogen, Waltham, MA, USA) vector to generate a pENTRY-VvMYBPA1 construct using BP Clonase (Invitrogen), which was then sequenced. The correct pENTRY-VvMYBPA1 plasmid was then cloned into a GATEWAY-compatible vector pB7FWG2 utilizing LR Clonase (Invitrogen). The plasmid pB7FWG2-VvMYBPA1 was further validated by restriction endonuclease digestion via *Xba* I and *Xho* I sites.

### 4.3. Generation of VvMYBPA1-Overexpressing Arabidopsis Transgenics

*Agrobacterium tumefaciens* strain GV3101 carrying the VvMYBPA1 overexpression vector was used to transform *A. thaliana* by the floral dip method [44]. T0 seeds were harvested and sown on half-strength MS medium supplemented with 10 mg/L Basta. Three independent overexpression lines (OE#1, OE#10, OE#23) were selected from the T1 population based on transgene expression stability, with homozygous T3 progenies serving as the experimental materials.

### 4.4. B. cinerea Infection Assay

*B. cinerea* strain B05.10, isolated from decayed grapefruits in our lab, was preserved on PDA plates (22 °C). For inoculation of *A. thaliana*, fresh *B. cinerea* conidial suspensions were adjusted to an inoculum density of 5 × 10^6^ conidia/mL as described by Xue and Yi [32]. For consistent infection, 10 μL inoculum droplets were applied to the abaxial surface of five mature leaves per plant at developmental stage 3.90 (4-week-old vegetative growth). For gene expression analysis, leaf samples were acquired at 0 and 72 h post inoculation (hpi). Histopathological evaluation was performed using trypan blue (cell viability), 3,3’-diaminobenzidine (DAB, H_2_O_2_ accumulation), and nitroblue tetrazolium (NBT, superoxide anion) staining at both pre-inoculation (0 hpi) and advanced infection (72 hpi) stages. For morphological assessment and lesion diameter measurement, detached leaves were infected with 10 µL of prepared conidial suspension using the droplet method. All measurements and photographic documentation were performed at 72 hpi.

For pathogen challenge assays, visually uniform ‘Kyoho’ fruits showing no signs of mechanical injury or pathological symptoms were subjected to biphasic surface decontamination (1% NaClO → 75% ethanol) with intermediate rinses in sterile ddH_2_O, and then air-drying at room temperature. Precise epidermal trauma (2 mm transverse × 4 mm longitudinal) was generated in the equatorial plane of each berry using sterile needles, and 10 μL of *B. cinerea* conidial suspensions (2 × 10^6^ conidia/mL) was inoculated into each wound. Post inoculation, berries were maintained in controlled conditions: primary incubation at 22 °C with saturated humidity (90–100% RH) under dark regime for 24 h, transitioning to diurnal cycles (12 h light/12 h dark) for disease progression monitoring. Three independent biological replicates were performed, each consisting of 10 berries. Pericarp tissues (approximately 1 cm in diameter) surrounding the inoculation site were used for physiological index determination and gene expression detection.

### 4.5. ROS Levels and Cell Death Assay

Hydrogen peroxide (H_2_O_2_) accumulation was detected using DAB staining [45]. Briefly, leaf samples were vacuum-infiltrated for 20 min in freshly prepared 1 mg/mL DAB solution and incubated in darkness overnight. Subsequently, leaf specimens underwent thermal destaining (100 °C, 10 min) in acetic acid: glycerol: ethanol (1:1:3, *v*/*v*/*v*), followed by immersion in 95% ethanol. Superoxide anion (O_2_^−^) accumulation was visualized by NBT staining [46]. Superoxide localization was achieved through vacuum-assisted infiltration (20 min, 25 inHg) of NBT reaction buffer (10 mM phosphate buffer (pH 7.8), 10 mM NaN_3_, 0.1% (*w*/*v*) NBT), with subsequent dark incubation (60 min, 22 °C). Stained tissues were cleared by thermal treatment (100 °C, 10 min) in destaining solvent (acetic acid: glycerol: ethanol, 1:1:3 *v*/*v*/*v*) and archived in 95% ethanol for microscopic analysis. Histochemical detection of compromised cells was conducted via trypan blue uptake assay, according to Vogel and Somerville [47]. All specimens were imaged under controlled bright-field conditions under an Olympus light microscope (Olympus Corporation, Tokyo, Japan). The content of H_2_O_2_ and malondialdehyde (MDA) was monitored according to Han et al. [48]. For each treatment, a minimum of six leaves per biological replicate were analyzed, with three independent biological replicates performed.

### 4.6. Histochemical Staining of DMACA

Proanthocyanidin localization in *A. thaliana* seeds was visualized through dimethylaminocinnamaldehyde (DMACA) histochemistry [12]. Seed specimens were immersed in freshly prepared DMACA reagent (1% *w*/*v* DMACA in 6 M HCl-methanol, 1:99 *v*/*v*) for 20 min (25 °C), followed by sequential ethanol washes (75% *v*/*v*, 4 × 5 min) to eliminate nonspecific staining. PA deposition patterns were documented using stereomicroscopy with consistent illumination settings.

### 4.7. RNA Isolation and RT-qPCR

Total RNA extraction from Arabidopsis leaves was conducted utilizing Trizol reagent (TaKaRa, Beijing, China). At the same time, grape berry pericarp RNA was isolated through an adapted CTAB-based approach [49]. RNA quality was assessed via 2.0% agarose gel electrophoresis (Sigma-Aldrich, Hong Kong, China), with quantification performed using a Bio Spectrometer fluorescence spectrophotometer (Eppendorf, Hamburg, Germany). cDNA synthesis was performed using the Prime Script™ RT reagent Kit with a gDNA Eraser (TaKaRa, Osaka, Japan). Gene-specific primers (Supplementary Table S1) were obtained from Sangon Biotech (Shanghai, China). Quantitative PCR was carried out on a Bio-Rad CFX96™ system under standardized thermal cycling parameters: following a 30 s hot start at 95 °C, the reaction mixture underwent 40 amplification cycles comprising 5 s denaturation pulses at 95 °C and 30 s incubation periods at 60 °C. For Arabidopsis and grapevine, gene expression levels were standardized against *AtActin2* and *Vvβactin* as internal controls, with the 2^−ΔΔCt^ method employed for comparative quantification [50].

### 4.8. Transient Overexpression of VvMYBPA1 in Grape Berries

Transient overexpression of VvMYBPA1 in grape berries was conducted following the methodology established by Xie et al. [51]. *Agrobacterium tumefaciens* strains harboring either the pB7FWG2-VvMYBPA1 construct or the empty pB7FWG2 vector were first grown on LB agar plates containing 25 mg·L⁻^1^ rifampicin and 50 mg·L⁻^1^ spectinomycin. For pre-culture preparation, a selected colony was introduced into 2 mL of liquid LB medium and cultured at 28 °C (220 rpm) overnight (12 h). The bacterial culture was subsequently expanded to 20 mL in LB medium and allowed to grow until the OD_600_ reached 0.6–0.8. Cell pellets obtained by centrifugation (5 min, 4000× *g*, 25 °C) were subsequently resuspended in infiltration buffer of identical volume containing magnesium chloride (10 mM) and acetyleugenone (200 µM). The suspension was conditioned at 28 °C for a duration of 3 to 4 h before being used for infiltration. Transient expression was achieved by microinjecting 300 μL of Agrobacterium suspension into the stylar apex of grape berries using a 1 mL syringe, ensuring minimal leakage. Berries were sampled at 4 days post infiltration (DPI) for RNA extraction. The expression levels of VvMYBPA1 were quantified at 0 and 4 DPI using the RT-qPCR protocol described earlier. The agroinfiltrated grape fruits were kept for four days before being subjected to pathogenic infection tests.

### 4.9. Quantification of Oligomeric Proanthocyanidins Content

Oligomeric proanthocyanidins content (OPC) was quantified using a plant OPC assay kit (Beijing Solarbio Technology Co., Ltd., Beijing, China) following the manufacturer’s protocol. A standard curve was established based on the linear relationship between OPC concentration and absorbance. The OPC concentration (X, mg·mL^−1^) was calculated using the linear regression equation X = (A_500_ − 0.0167)/0.1829 (R^2^ = 0.9981), where A_500_ represents the absorbance at 500 nm.

Grape fruit samples were freeze-dried to constant weight, homogenized using a mortar, and precisely weighed (0.1 g). The absorbance was recorded at 500 nm following the manufacturer’s protocol. OPC content was determined by normalizing the standard curve-derived concentration (X) against sample dry mass (W), expressed as mg per gram dry weight (mg·g^−1^ DW).

### 4.10. Measurement of Enzyme Activity

The enzymatic activities of four defense-related proteins—phenylalanine ammonia-lyase (PAL), β-1,3-glucanase (BGL), chitinase (CHI), and polyphenol oxidase (PPO)—were assayed following established protocols [32]. Concurrently, antioxidant enzyme activities (catalase (CAT), peroxidase (POD), and superoxide dismutase (SOD)) were evaluated according to the methodology of Jiang et al. [35].

### 4.11. Statistical Analysis

Three independent biological replicates were included for each experimental treatment. Results are presented as mean values ± standard error (SE). For transgenic Arabidopsis lines, statistical comparisons of lesion diameter measurements, transcriptional profiles, enzymatic activities, and secondary metabolite accumulation were conducted using two-tailed paired Student’s *t*-tests, with asterisks indicating significance levels (* *p* < 0.05; ** *p* < 0.01). For agro-infiltrated grape samples, one-way analysis of variance (ANOVA) was applied, with Duncan’s multiple range test used for comparisons (*p* < 0.05, denoted by different letters). Data processing was conducted using Microsoft Excel (v2019, Microsoft Corp.). Statistical analyses were conducted using SPSS Statistics 16.0, while data visualization was implemented in GraphPad Prism 6.

## Data Availability

The original contributions presented in this study are included in the article/Supplementary Materials. Further inquiries can be directed to the corresponding authors.

## Supplementary Materials

The following supporting information can be downloaded at: https://www.mdpi.com/article/10.3390/plants14162469/s1, Table S1: List of primers used in this study. Figure S1. Expression patterns of *VvMYBPA1* in grapevine cultivars responding to *Botrytis cinerea* infection.
