# Supplementary material for: Transient Overexpression of VvMYBPA1 in Grape Berries Enhances Susceptibility to Botrytis cinerea Through ROS Homeostasis Modulation
Source: Plants (Basel). 2025 Aug 9;14(16):2469. doi: 10.3390/plants14162469 (PMC12389462; doi:10.3390/plants14162469)
Supplement: Supplementary file 1 [file plants-14-02469-s001.zip › supplemental files/Supplemental table 1.docx]

Table S1. List of primers used in this study.

| **Primer name** | **Gene ID** | **Primer sequence（5’-3’）** |
| --- | --- | --- |
| VvMYBPA1 CDS F | Vitvi15g00938 | GGGGACAAGTTTGTACAAAAAAGCAGGCTTAATGGGCAGAGCACCTTGT |
| VvMYBPA1 CDS R |  | GGGGACCACTTTGTACAAGAAAGCTGGGTAAATGAGTAGTGATTCGGC |
| AtTUB2-F | AT5G62690 | GTTCTCGATGTTGTTCGTAAG |
| AtTUB2-R |  | TGTAAGGCTCAACCACAGTAT |
| AtActin2-F | AT3G18780 | AGTGTCTGGATCGGTGGTTC |
| AtActin2-R |  | CCCCAGCTTTTTAAGCCTTT |
| B.cActin-F | BCIN_16g02020 | GCTGGTCGTGATTTGACTGAT |
| B.cActin-R |  | GACTGGCGGTTTGGATTTCTT |
| Vvβactin-F | Vitvi04g01613 | CTTGCATCCCTCAGCACCTT |
| Vvβactin-R |  | TCCTGTGGACAATGGATGGA |
| VvMYBPA1-F | Vitvi15g00938 | CTCTCCCCAAGAAAGCTGGT |
| VvMYBPA1-R |  | TTCGACCTGGAAGCCTACCT |
| AtRBOHD-F | AT5G47910 | ACGTGCGTCCAAGAAAAACG |
| AtRBOHD-R |  | CGTAAGAAGGGCTAGCTCCG |
| AtRBOHF-F | AT1G64060 | TCAGAGCCGACGAAACAACA  qRT-AtRBOHF R TCCGAGATCGAATCCGCATG |
| AtRBOHF-R |  | TCCGAGATCGAATCCGCATG |
| VvRBOHA-F | Vitvi02g00048 | ATGATGTCCTGCTACTTGTTGG |
| VvRBOHA-R |  | TCTGATACTGAATCCTGCTCCT |
| VvRBOHB-F | Vitvi14g00183 | GGAGTGTGATGAATGAGGTGAC |
| VvRBOHB-R |  | TGGCGTGATGGAGTGATTGA |
| VvVPE-F | Vitvi02g00505 | AAATTACAGGCACCAGGCTGA |
| VvVPE-R |  | ACACATCATCACCTTGCGGAT |
| VvSOD-F | Vitvi14g02629 | GTAATGAGGGTGTTTGTGG |
| VvSOD-R |  | TTCTCGTCTTCAGGAGCA |
| VvPOD-F | Vitvi08g00097 | AGATGGCTCAGCAAGTGGTC |
| VvPOD-R |  | GTCAGCGCAGGAAACAACAG |
| VvCAT-F | Vitvi18g00095 | TGTTGGAAGAAGAGGCAATCAG  VvCAT-R GACACCAGGAACCACAATAGC |
| VvCAT-R |  | GACACCAGGAACCACAATAGC |
