# Supplementary material for: Transient Overexpression of VvMYBPA1 in Grape Berries Enhances Susceptibility to Botrytis cinerea Through ROS Homeostasis Modulation
Source: Plants (Basel). 2025 Aug 9;14(16):2469. doi: 10.3390/plants14162469 (PMC12389462; doi:10.3390/plants14162469)
Supplement: Supplementary file 1 [file plants-14-02469-s001.zip › supplemental files/Supplemental table 1.pdf]

Table S1. List of primers used in this study.

Primer name	Gene ID	Primer sequence（5′-3′）
VvMYBPA1 CDS F	Vitvi15g00938	GGGGACAAGTTTGTACAAAAAAGCAGGCTTAATGGGCAGAGCACCTTGT
VvMYBPA1 CDS R		GGGGACCACTTTGTACAAGAAAGCTGGGTAAATGAGTAGTGATTCGGC
AtTUB2-F	AT5G62690	GTTCTCGATGTTGTTCGTAAG
AtTUB2-R		TGTAAGGCTCAACCACAGTAT
AtActin2-F	AT3G18780	AGTGTCTGGATCGGTGGTTC
AtActin2-R		CCCCAGCTTTTTAAGCCTTT
B.cActin-F	BCIN_16g02020	GCTGGTCGTGATTTGACTGAT
B.cActin-R		GACTGGCGGTTTGGATTTCTT
Vvβactin-F	Vitvi04g01613	CTTGCATCCCTCAGCACCTT
Vvβactin-R		TCCTGTGGACAATGGATGGA
VvMYBPA1-F	Vitvi15g00938	CTCTCCCCAAGAAAGCTGGT
VvMYBPA1-R		TTCGACCTGGAAGCCTACCT
AtRBOHD-F	AT5G47910	ACGTGCGTCCAAGAAAAACG
AtRBOHD-R		CGTAAGAAGGGCTAGCTCCG
AtRBOHF-F	AT1G64060	TCAGAGCCGACGAAACAACA
AtRBOHF-R		TCCGAGATCGAATCCGCATG
VvRBOHA-F	Vitvi02g00048	ATGATGTCCTGCTACTTGTTGG
VvRBOHA-R		TCTGATACTGAATCCTGCTCCT
VvRBOHB-F	Vitvi14g00183	GGAGTGTGATGAATGAGGTGAC
VvRBOHB-R		TGGCGTGATGGAGTGATTGA
VvVPE-F	Vitvi02g00505	AAATTACAGGCACCAGGCTGA
VvVPE-R		ACACATCATCACCTTGCGGAT
VvSOD-F	Vitvi14g02629	GTAATGAGGGTGTTTGTGG
VvSOD-R		TTCTCGTCTTCAGGAGCA
VvPOD-F	Vitvi08g00097	AGATGGCTCAGCAAGTGGTC
VvPOD-R		GTCAGCGCAGGAAACAACAG
VvCAT-F	Vitvi18g00095	TGTTGGAAGAAGAGGCAATCAG
VvCAT-R		GACACCAGGAACCACAATAGC
