# Supplementary figures and images for: Transient Overexpression of VvMYBPA1 in Grape Berries Enhances Susceptibility to Botrytis cinerea Through ROS Homeostasis Modulation
Source: Plants (Basel). 2025 Aug 9;14(16):2469. doi: 10.3390/plants14162469 (PMC12389462; doi:10.3390/plants14162469)

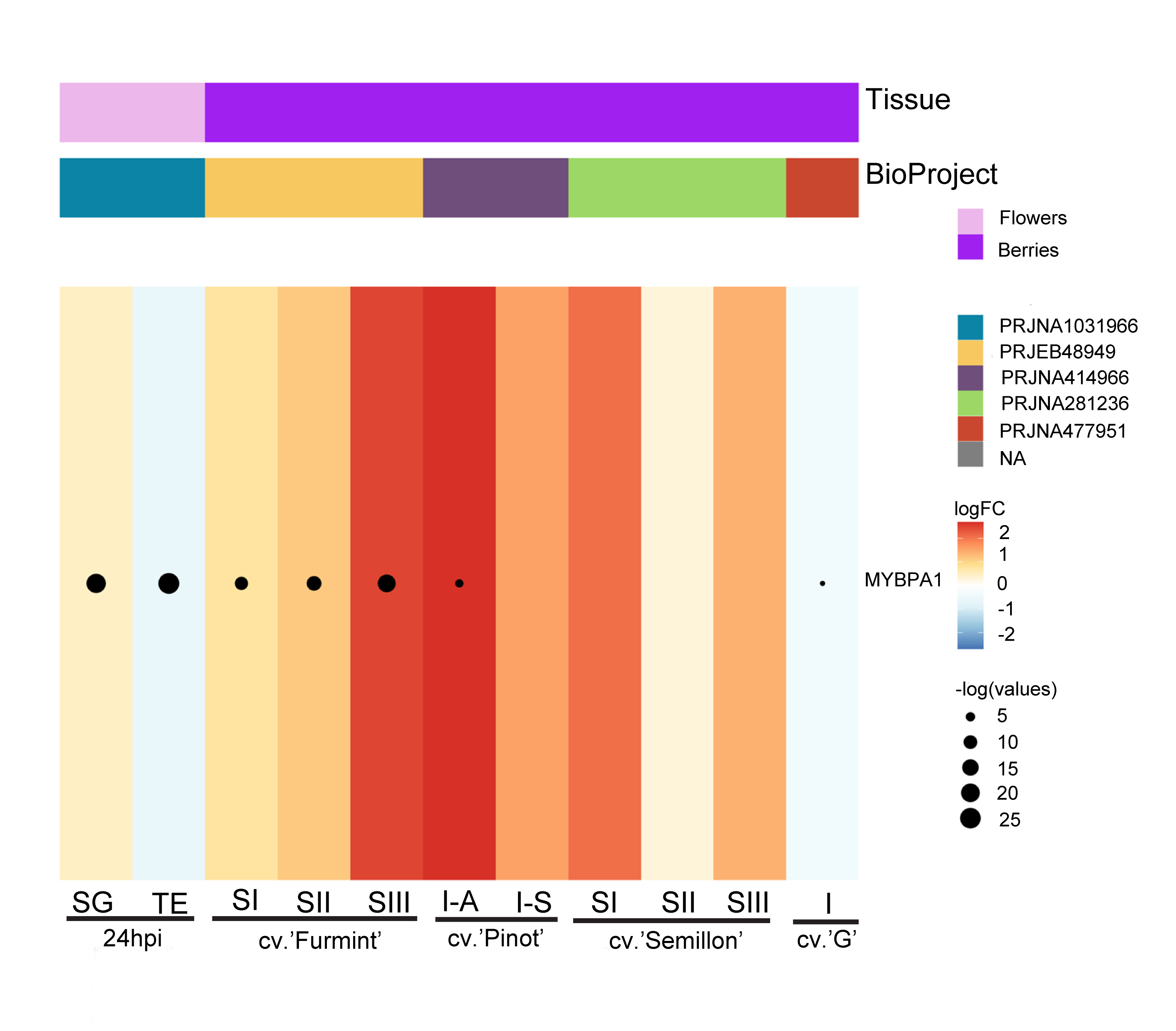

Supplement: Supplementary file 1 [file plants-14-02469-s001.zip › supplemental files/Figure S1.tif]
